# Spontaneous Idiopathic Non-venereal Cavernosal Abscess: A Rare Case Report and Review of Literature

**DOI:** 10.7759/cureus.92612

**Published:** 2025-09-18

**Authors:** Prajeeth Reddy, Punith Jain R, Hariharasudhan Sekar, Velmurugan Palaniyandi, Sriram Krishnamoorthy

**Affiliations:** 1 Urology, Sri Ramachandra Institute of Higher Education and Research, Chennai, IND; 2 Urology and Renal Transplantation, Sri Ramachandra Institute of Higher Education and Research, Chennai, IND

**Keywords:** acinetobacter wolffii, antibiotics, cavernosal abscess, erythema nodosum, penile abscess

## Abstract

Penile abscesses are uncommon in urology and usually arise from trauma, intracavernous injections, systemic infections, and sexually transmitted infections (STIs). However, spontaneous cases with no definitive causes are sporadic. Here, we describe an unusual presentation in a 55-year-old man with no history of trauma, diabetes, immunosuppression, or recent sexual activity. During this time, he was being treated for erythema nodosum when he developed worsening penile swelling and pain.

Imaging (ultrasound and MRI) showed a lesion within the corpora cavernosa; surgical exploration and biopsy confirmed the presence of cavernous abscess, and the pus culture grew Acinetobacter wolffi, an organism not usually associated with penile infections. Surgically, the abscess was drained, and antibiotics were administered based on sensitivities. The patient made a good recovery without any residual symptoms, and follow-up demonstrated no effect on erectile function or scarring or curvature (both of which can be significant concerns in these cases).

This case highlights the diagnostic challenges of cavernosal abscesses, particularly in the absence of typical risk factors. Early imaging and surgical intervention were crucial for a positive outcome. Urologists and primary care providers should maintain a high suspicion for such atypical presentations when evaluating unexplained penile swelling. To our knowledge, this is among the first reported cases of a spontaneous Acinetobacter wolffii-induced cavernosal abscess.

## Introduction

Penile abscess is an uncommon but potentially serious urological condition characterized by a localized infection and the accumulation of pus within the penile tissues. Most cases documented in the literature are associated with identifiable risk factors, including trauma, priapism, intracavernous injections for erectile dysfunction, hematogenous spread from distant infections (such as dental or perianal abscesses), or sexually transmitted infections [1]. Cavernosal abscesses are particularly rare, and spontaneous or idiopathic cases are only sporadically reported.

The clinical presentation of a penile abscess typically includes pain, swelling, and erythema, and may be accompanied by systemic symptoms such as fever or malaise. Immunocompromised individuals - especially those with diabetes mellitus or HIV - are at greater risk due to compromised local immune defences and microvascular dysfunction [2]. The most frequently isolated organisms from abscess cultures include "Staphylococcus aureus", "Streptococcus species", "Escherichia coli", and anaerobes such as "Bacteroides" [3].

Although the diagnosis tends to be clinical, imaging studies help determine the extent of the abscess and differentiate it from other lesions on the penis (e.g., neoplasms or hematomas). Ultrasonography, CT, or MRI can aid in this process. Ultrasound-guided aspiration is also helpful for both diagnostic confirmation and initial therapeutic drainage. Incision and drainage, along with systemic antibiotic therapy based on culture and sensitivity results, are usually definitive; if treatment is delayed or suboptimal, complications (e.g., erectile dysfunction, penile curvature, or fibrosis) may develop. In this report, we describe a rare case of spontaneous idiopathic cavernosal abscess in a middle-aged male without any identifiable predisposing factors, who was concurrently undergoing treatment for erythema nodosum. Through this case, we aim to contribute to the limited body of literature on this unusual clinical entity and emphasize the importance of considering penile abscesses, even when traditional risk factors are absent.

## Case presentation

**Figure 1:**
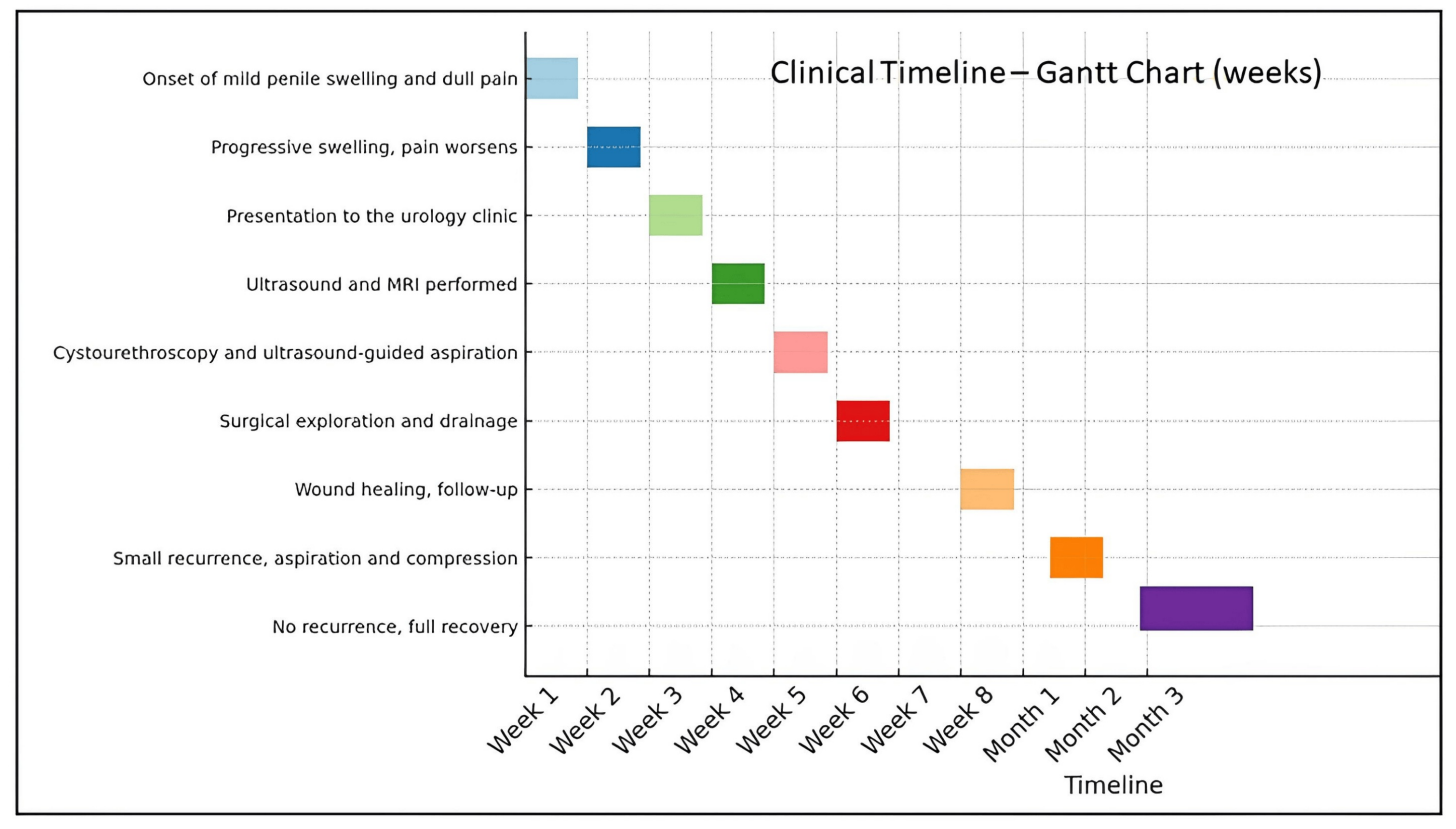
Timeline of events

A 55-year-old man reported to the outpatient urology clinic with a two-week history of gradual swelling of his penis and dull pain at the base of the penis. His symptoms progressed from an insidious onset until they became mildly uncomfortable during micturition, and he noticed decreased libido. He denied fever, rigors, trauma, penile instrumentation, urethral catheterization, or recent sexual activity. He was not found to have diabetes mellitus, hypertension, tuberculosis, HIV, or other immunocompromising conditions in his medical history; however, he had been on corticosteroid therapy for erythema nodosum, which began approximately four months prior (Figure 1).

On general examination, the patient was afebrile and hemodynamically stable. Systemic examination was unremarkable. Local genital examination revealed a non-tender, fluctuant swelling measuring approximately 2-3 cm at the dorsal base of the penis, predominantly affecting the left corpus cavernosum. The overlying skin was intact, with no signs of ulceration or erythema. There was no inguinal lymphadenopathy. Digital rectal examination and prostate assessment were within normal limits.

Laboratory investigations, including a complete blood count, renal function tests, liver enzymes, and fasting blood glucose, were within normal ranges. Inflammatory markers were mildly elevated. Serologic tests for HIV, VDRL, and hepatitis B/C were negative. TB Quantiferon Gold was also negative (Table 1).

**Table 1:** Laboratory Investigations BUN: Blood urea nitrogen; VDRL: Venereal disease research laboratory; SGOT: Serum Glutamic Oxaloacetic Transaminase; SGPT: Serum Glutamic Pyruvic Transaminase

Parameter	Values	Reference Range
Hemoglobin	13 gm/dL	13-17 gm/dL
Total Counts	9100 cells/cumm	4000-11000 cells/cumm
Neutrophils	59.1%	45-70%
Lymphocytes	31.6%	25-40%
Eosinophils	2.1%	1-6%
Platelet count	2.44 lakhs/cumm	1.5-4.5 lakhs/cumm
BUN	8 mg/dL	7.9-20.1 mg/dL
Creatinine	1.1 mg/dL	0.8-1.3 mg/dL
Fasting blood glucose	89 mg/dL	70-100 mg/dL
C-Reactive Protein	10.2 mg/L	0.3-6.0 mg/L
Total Bilirubin	0.39 mg/dL	<1.2 mg/dL
Direct Bilirubin	0.20 mg/dL	<0.30 mg/dL
Alk Phosphatase	97 IU/L	32-120 IU/L
SGOT	16 IU/L	<40 IU/L
SGPT	15 IU/L	<41 IU/L
HIV	Negative	
VDRL	Negative	
Hepatitis B/C	Negative	
TB Quantiferon Gold	Negative	

**Figure 2:**
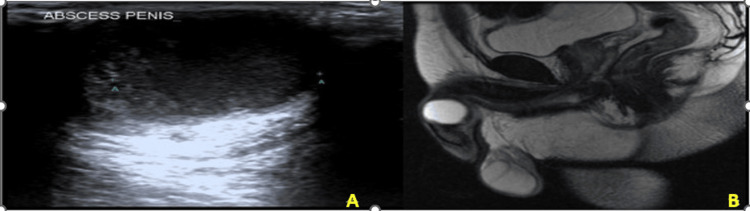
Imaging Representation of Penile Abscess (A) Ultrasound image showing a well-defined fluid collection of size 3.0 x 2.0 x 2.7 cm predominantly on the left side of the cavernosa. (B) MRI showing a well-defined lesion with stretching of the tunica albuginea and displacing the spongiosum.

Penile ultrasonography revealed a well-defined 3×3 cm hypoechoic lesion with internal echoes and peripheral vascularity localized to the left corpus cavernosum (Figure 2A). MRI of the pelvis confirmed a 3×2.5 cm mass lesion with rim enhancement and features suggestive of a well-encapsulated abscess. However, a smooth muscle tumor was initially considered in the differential diagnosis (Figure 2B).

Cystourethroscopy was performed to exclude urethral pathology and revealed no abnormalities in the anterior or posterior urethra or bladder. Subsequently, under sterile conditions and anaesthesia, ultrasound-guided aspiration was attempted, yielding approximately 10 mL of thick, purulent material, which was sent for culture and sensitivity. Due to persistent induration and suspicion of solid components, a decision was made to proceed with surgical exploration.

**Figure 3:**
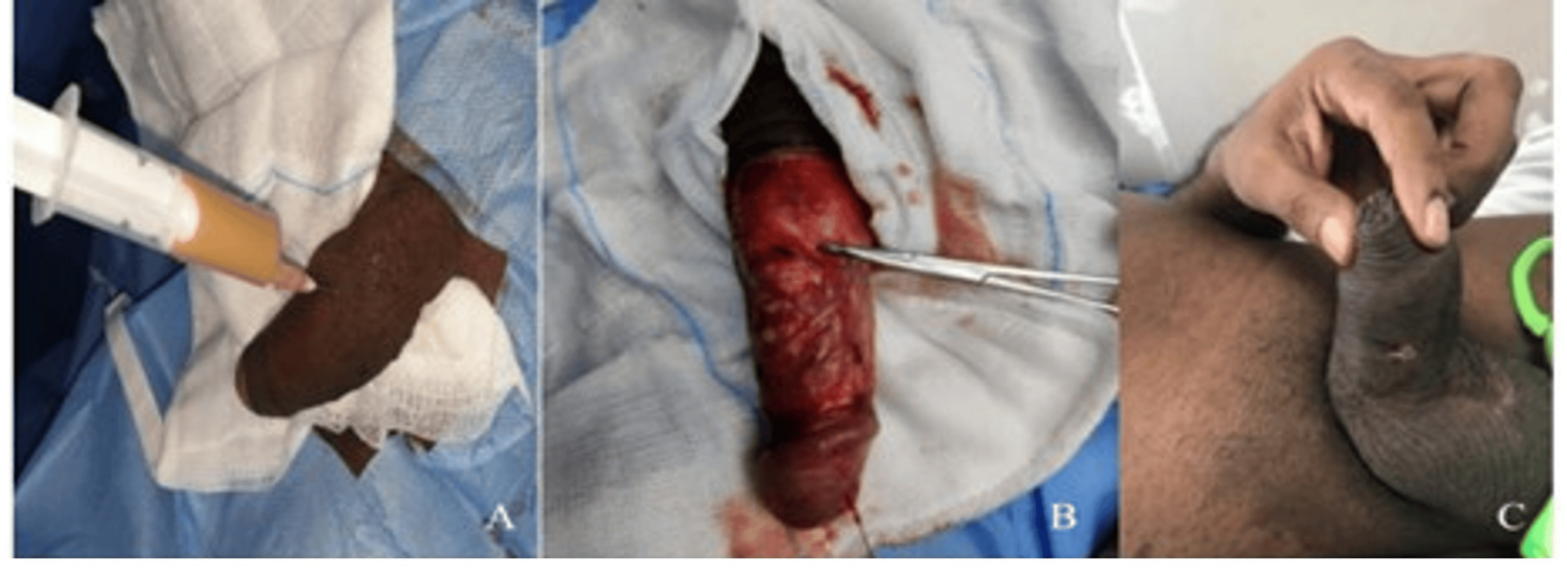
Interventions and Post Operative Image of Penile Abscess (A) Needle aspiration of purulent pus. (B) De-gloved penis before incisional biopsy and pus drainage. (C) Post-operative review after one week.

A circumferential penile degloving was performed. Intraoperatively, extensive induration was noted extending from the left corpus cavernosum toward the contralateral side. Thorough drainage and irrigation were carried out. A biopsy of the indurated tissue was sent for histopathological evaluation, and the corpora were closed with 4-0 polydioxanone (PDS) sutures. The wound was closed in layers (Figure 3A-3C).

**Figure 4:**
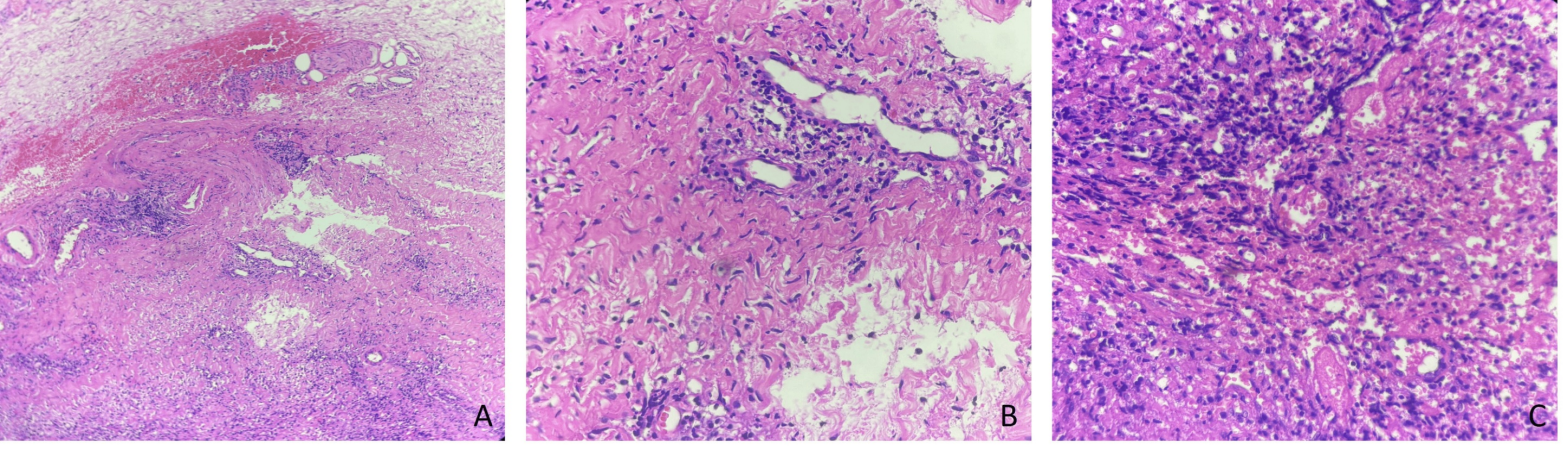
Histopathology (A) H&E, under 200x magnification, showing fibrosis with chronic inflammatory infiltrate and focal haemorrhage. (B) H&E, under 200x magnification, showing fibrosis with perivascular chronic inflammatory infiltrate. (C) H&E, under 400x magnification, showing dense neutrophilic aggregates, interspersed neutrophils in the background of necrosis.

**Figure 5:**
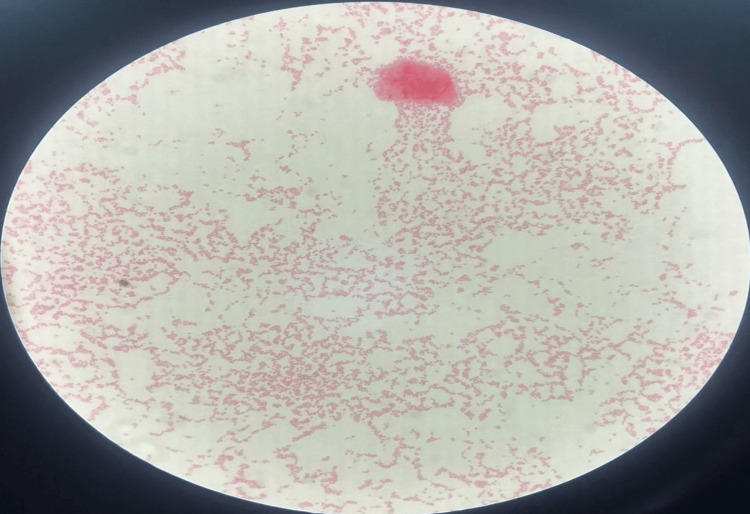
Microscopic Examination of Pus Culture Gram staining showing Gram-negative coccobacilli, suggesting Acinetobacter.

Postoperatively, the patient was managed with broad-spectrum intravenous antibiotics and analgesia. Histopathology showed acute and chronic inflammatory infiltrates with areas of necrosis, confirming abscess formation without any evidence of malignancy (Figure 4A-4C). Gram stain revealed a few Gram-negative coccobacilli, as well as budding yeast, and acid-fast bacilli staining was negative. Culture and sensitivity from the aspirated pus done on MacConkey agar identified "Acinetobacter wolffii" (Figure 5). Antibiotic therapy was switched to oral amoxicillin-clavulanate based on the results of sensitivity testing.

The patient responded well to treatment. At two-week follow-up, the wound had healed satisfactorily, with minimal residual induration and no evidence of penile curvature or erectile dysfunction. At one-month follow-up, a small recurrent collection was aspirated under aseptic precautions, and a compression dressing was applied. On long-term follow-up, the patient remained asymptomatic with normal urinary flow and satisfactory sexual function.

## Discussion

Penile abscesses are rare clinical entities, and cavernosal abscesses are extremely rare; their etiology is often secondary to identifiable risk factors (trauma, intracavernosal injections, systemic infections [4-7], tuberculous cold abscess of the corpus cavernosum [8,9], and perineal or anorectal abscesses [10]) with reports linking penile abscesses to sexually transmitted infections in immunocompromised patients. The paired erectile body, the corpus cavernosum, is usually resistant to infection because of its dense tunica albuginea; however, direct trauma and hematogenous spread of bacteria can cause infection. In cases like the one described here, with spontaneous idiopathic causes, there are no identifiable risk factors.

The patient described in this case did not present with the typical risk factors commonly associated with cavernosal abscesses. He had no history of trauma, urological procedures, diabetes, or known immunosuppression. His only notable medical history was erythema nodosum - an inflammatory condition often linked to systemic disease or medication exposure, which may have subtly influenced his local immune response.

Acinetobacter wolffii is a rare Gram-negative coccobacillus, typically considered an environmental or opportunistic pathogen, more frequently encountered in intensive care settings or among immunocompromised individuals. It is not commonly isolated from soft tissue infections. To the best of our knowledge, this is the first reported case in which "Acinetobacter wolffi" was isolated from a cavernosal abscess.

This case highlights that the pathogens causing rare infections are evolving. Therefore, culture-directed antibiotic therapy remains crucial, as clinicians should be vigilant for unusual organisms in atypical clinical presentations. Diagnostic imaging was also very critical to this evaluation. Ultrasonography initially revealed a fluid-filled collection with internal echoes, which led to the diagnosis and aspiration. Magnetic resonance imaging (MRI) further differentiated the lesion from potential malignancy by showing rim enhancement and internal necrosis consistent with abscess formation.

The role of cystoscopy in this case was to exclude any urethral involvement, which can sometimes coexist or serve as a source of infection.

Treatment options for penile abscesses include conservative management with image-guided aspiration and antibiotics, as well as open surgical drainage. While minimally invasive procedures are attractive due to reduced postoperative morbidity, they carry the risk of incomplete evacuation and recurrence [11]. Open surgical drainage, as used in this case, enables the complete evacuation of abscesses and tissue biopsy to rule out underlying malignancy, particularly when imaging is inconclusive. Complications associated with open drainage include penile curvature, erectile dysfunction, and fibrosis due to scar formation [12]. Fortunately, our patient did not experience any of these sequelae.

Penile abscesses have been reported in a variety of organisms; however, literature reports include Staphylococcus aureus, Streptococcus anginosus, Mycobacterium tuberculosis, Actinomyces species, and various enteric Gram-negative bacteria. The isolation of Acinetobacter wolffii in this case contributes to the growing list of possible pathogens and underscores the necessity for culture-directed antibiotic therapy. Long-term prognosis is dependent on early recognition and intervention, as well as follow-up, because delayed or inadequate treatment can result in life-threatening complications such as necrotizing fasciitis or sepsis. Therefore, clinicians must remain aware of this possibility when faced with unexplained penile swelling, even in the absence of common risk factors.

This case was complicated by the fact that spontaneous idiopathic cavernosal abscesses are very uncommon, and in a patient without any of the common risk factors, it is difficult to diagnose. The infection was successfully managed using imaging, surgical evaluation, microbiological testing, and histopathological confirmation. The isolation of Acinetobacter wolffii as the causative agent reinforces the growing awareness of various pathogens implicated and supports the need for antibiotic therapy guided by culture results. Although conservative measures can be tried, surgical drainage is typically the mainstay of treatment (especially if imaging is non-contributory or aspiration is not productive).

## Conclusions

Early recognition and prompt intervention are key to preventing complications such as penile curvature, fibrosis, or long-term erectile dysfunction; therefore, clinicians should keep this unusual condition in mind when examining patients presenting with unexplained swelling or masses of the penis.

## References

[1] Moemen MN, Hamed HA, Kamel II, Shamloul RM, Ghanem HM (2004). Clinical and sonographic assessment of the side effects of intracavernous injection of vasoactive substances. Int J Impot Res.

[2] Dugdale CM, Tompkins AJ, Reece RM, Gardner AF (2013). Cavernosal abscess due to Streptococcus anginosus: a case report and comprehensive review of the literature. Curr Urol.

[3] Garcia C, Winter M, Chalasani V, Dean T (2014). Penile abscess: a case report and review of literature. Urol Case Rep.

[4] Pearle MS, Wendel EF (1993). Necrotizing cavernositis secondary to periodontal abscess. J Urol.

[5] Fernández Goméz JM, Regadera Sejas FJ, Pérez García FJ, Sahagun Arguello JL (1997). Absceso bilateral de cuerpos cavernosos [Bilateral abscess of cavernous bodies]. Actas Urol Esp.

[6] Sater AA, Vandendris M (1989). Abscess of corpus cavernosum. J Urol.

[7] Thanos L, Tsagouli P, Eukarpidis T, Mpouhra K, Kelekis D (2011). Computed tomography-guided drainage of a corpus cavernosum abscess: a minimally invasive successful treatment. Cardiovasc Intervent Radiol.

[8] Yachia D, Friedman M, Auslaender L (1990). Tuberculous cold abscess of the corpus cavernosum: a case report. J Urol.

[9] Murali TR, Raja NS (1998). Cavernosal cold abscess: a rare cause of impotence. Br J Urol.

[10] Sivaprasad G, Devanathan KS, Ganesh G (2005). Corpora cavernositis caused by Actinomycetes. Scand J Urol Nephrol.

[11] Pascual Regueiro D, García de Jalón Martínez A, Mallén Mateo E, Sancho Serrano C, Borque Fernando A, Rioja Sanz LA (2003). Incurvación peneana secundaria a absceso de cuerpo cavernoso [Penile curvature secondary to cavernous body abscess]. Actas Urol Esp.

[12] Niedrach WL, Lerner RM, Linke CA (1989). Penile abscess involving the corpus cavernosum: a case report. J Urol.

